# Learning Curve of Closed Reduction and Internal Fixation for Supracondylar Fractures of the Humerus in Children

**DOI:** 10.3389/fped.2022.945616

**Published:** 2022-07-07

**Authors:** Chuang Qian, Yiming Zheng, Junrong Meng, Yueqiang Mo, Jinhua Sun, Hao Li, Dahui Wang

**Affiliations:** ^1^Department of Orthopedics, Children's Hospital of Fudan University, National Children's Medical Center, Shanghai, China; ^2^Department of Neurosurgery, Children's Hospital of Fudan University, National Children's Medical Center, Shanghai, China; ^3^Shanghai Mental Health Center, Shanghai, China

**Keywords:** supracondylar fractures of the humerus, closed reduction, learning curve, specialist training, functional recovery

## Abstract

**Background:**

This study aimed to identify the threshold for success in supracondylar humeral fracture surgery by describing the learning curve for beginners and exploring the relationship between the learning curve and the prognosis of supracondylar fractures of the humerus.

**Methods:**

Surgical information was collected of the first 100 humeral fractures treated by four pediatric orthopedic surgeons. The relationship between operation time, wire placement success rate, and surgical experience was determined using the restricted cubic strip (RCS). The inflection point in the curve and other risk factors that may affect fracture prognosis were collected and subjected to multiple logistic regression to clarify the relationship between the learning curve and prognosis of supracondylar fractures of the humerus. After the training, the four fresh surgeons were interviewed in the form of questionnaires to get feedback from the trainees.

**Results:**

A total of 400 supracondylar fractures of the humerus from four pediatric orthopedists were included in the study. On an RCS analysis, 65 surgical experiences were the inflection point of the learning curve. Before and after these 65 surgical experiences, there were significant differences in the patients' anatomical reduction (186 vs. 122, *P* < 0.001), conversion to incision (33 vs. 6, *P* = 0.008), and supervising physician guidance (28 vs. 2, *P* < 0.001). In the multiple logistic regression analysis, functional recovery after supracondylar fractures of the humerus was significantly associated with surgical experience, intraoperative conversion to incision, and post-operative infection. Four surgeons and a supervisor were interviewed. They believed that self-confidence establishment requires the experience accumulation of about 30 operations. The most critical surgical technique is the reduction of fractures.

**Conclusions:**

Although the accumulated experience of 30 operations can establish the self-confidence of trainers, fresh surgeons must accumulate experience with 65 operations to master closed reduction and internal fixation for supracondylar fractures. Surgical experience significantly impacts the post-operative recovery of patients with fractures.

**Level of Evidence:**

Level III.

## Introduction

Just as pilots learn to fly an aircraft, a learning curve is required for surgeons to master surgical skills. Pilots can prove mastery of technology by accumulating simulated flight time and completing mock exams, and airlines can determine the practice time required for pilots to master various aircrafts. However, surgeons cannot test their skills by collecting experiences with real patients. Before becoming proficient in surgical techniques, the medical team must give novice surgeons knowledge and technical support in practice and bear the medical risks of surgical failure. At present, the research on surgical learning curve often focuses on innovative surgery, difficult surgery and robotic surgery (1, 2). In orthopedics, there was also a large number of research on the learning curve of spine, pelvic fracture, congenital hip dysplasia and arthroscopic (3–5). There was no report on the learning curve for more elementary surgery. Thus, we asked, is it possible to change the way of thinking, starting with simple routine surgery, and explore a method of accurately calculating the learning curve?

Supracondylar fractures of the humerus account for two-thirds of all elbow injuries in children requiring surgery (6). Closed reduction and internal fixation of fractures with a Kirschner wire are not difficult, with minimal damage, fewer incisions, and accelerated healing (6). It should involve surgical skills that fresh pediatric orthopedists can master quickly so that pediatric orthopedists can build confidence in surgical success and lay the foundation for subsequent difficult operations.

This study aimed to explore the learning curve characteristics of pediatric fracture surgery by collecting the experience of four pediatric orthopedic surgeons in our center using surgeries treating supracondylar fractures, determining the learning threshold point, and exploring the relationship between the learning curve and prognosis of supracondylar fractures of the humerus.

## Materials and Methods

### Surgeon Selection

The surgeons were the four residents of the hospital, all of whom had completed 2 years of pediatric orthopedic resident training. During training, they were required to master all the methods of diagnosing and treating pediatric orthopedic-related diseases and were required to study all aspects of pediatric orthopedic-related surgical techniques as an assistant. After training, they began to perform common orthopedic surgeries in children.

### Patient Selection

The study included the first 100 patients with supracondylar fractures of the humerus that the above four residents were the chief surgeons in chronological order. Patients with multiple fractures, refractures, pathological fractures, congenital bone disease, neurovascular-related complications, and those assessed by the department requiring treatment by a more experienced physician were excluded.

### Surgical Plan Formulation and Implementation

The diagnosis of the patient's disease and the formulation of the surgical plan were jointly formulated by all orthopedic surgeons in the center after discussion. After confirming that the chief surgeon was one of the new residents mentioned above, the center designated an supervising doctor for the patient to prepare in the ward so that he/she could take over at any time in the event of an accident. Moreover, at the end of the operation, the supervising doctor confirmed the fracture reduction and internal fixation position on an intraoperative radiograph.

### Surgical Procedure

We will give priority to the closed reduction to correct the dislocated supracondylar fracture. When the closed reduction failed, the chief surgeon will switch to open reduction with the permission of the supervising doctor. Different K-wire arrangement schemes will be adopted according to the stability of the fracture (sector fixation with 2–3 Kirschner wires on the radial side or cross fixation with 2 Kirschner wires on the radial side and one Kirschner wire on the ulnar side) (7). After internal fixation, the supervising doctor will evaluate the position of fracture and internal fixation through observation in the operating room and intraoperative radiography (Due to the excellent shaping ability of supracondylar fracture, we do not require anatomical reduction of fracture at this time). Finally, we cut and bend the tail of the Kirschner wire, wrap it with gauze and leave it outside the skin.

We recorded each patient's operation time and the Kirschner wire placement success rate after each operation, and these two data were the main exposure variables for further research. We also recorded whether each operation was converted to open reduction during the operation and whether the intraoperative guidance of the supervising doctor was requested. Post-operatively, radiographs of the elbow joint were used to assess whether the fracture was anatomically reduced.

### Post-operative Complications

The supervising surgeon and the chief surgeon performed regular outpatient follow-up (every 2 weeks) for all cases. Fracture healing, wound infection, neurovascular function, and internal fixation loosening were evaluated and recorded at each follow-up visit.

### Prognosis

When a patient's fracture healed, the K-wire was removed and elbow joint function was exercised at home under the guidance of the chief surgeon. We evaluated and recorded the range of flexion and extension of the elbow joint 1 month after removal of the internal fixation. We considered the patient's recovery satisfactory if the elbow flexion and extension range of motion reached 80% of normal. If recovery was poor, we provided the patient with further rehabilitation guidance.

### Definitions and Standards for the Collected Data

Operation time: The operation time includes the whole process of disinfection, operation and wound dressing. It does not include the duration of induced anesthesia and anesthesia recovery.

Wire placement success rate: Number of K-wire used/ number of internal fixed attempts.

Anatomical reduction: The deformity and displacement of the fracture were completely corrected and the normal anatomy of the humerus was restored.

Functional reduction: The fracture was not completely reduced. We can accept a certain degree of rotation or radial displacement. However, on lateral radiographs, anterior humeral line must contact the capitulum humeri. Significant rotational displacement, ulnar displacement, and absence of contact with the humeral capitulum at anterior humeral line are not acceptable.

Infection: Including osteomyelitis, cellulitis and skin infection.

Fracture union: No local tenderness and abnormal activity; X-ray showed that the fracture line was fuzzy and there was continuous callus passing through the fracture line.

Delayed union: The fracture do not reach the union standard within 2 months after operation.

Implant related complications: Including internal fixation looseness and fracture displacement caused by internal fixation looseness.

Elbow function recovery: The range of motion of the normal elbow is about 135°-150°. We will take the patient's healthy elbow as the control. When the range of motion of the injured side reaches 80% of the control side, we will define it as good recovery.

### Statistical Analysis

We collected the data recorded above as well as the demographic and comorbidity variables for each patient, including age, sex, affected side (left or right), and fracture classification (8).

We used restricted cubic splines with four knots (9) to model the relationship between surgical experience, surgical time, and wire placement success rate after the adjustment for fracture classification, age, sex, and affected side (left or right). We examined the non-linear relationship between surgical experience and operative time and the wire placement success rate to identify any inflection point that could be used to dichotomize operative experience into categories in a clinically meaningful way. Once a reasonable inflection point was identified, the differences in the various data before and after the inflection point were used to verify its accuracy. Subsequently, we used multivariable logistic regression to determine the area under the curve for the models relating various surgical experience cutoff points to functional recovery. The surgical experience with the maximum area under the curve was selected as the cutoff point to dichotomize the surgical experience.

The final multivariate models were constructed in a stepwise backward manner. The model initially included all independent variables and sequentially excluded the variable with the highest *P*-value until only those of *P* < 0.20 remained. Values of *P* < 0.05 were considered statistically significant. Variables with *P*-values of 0.05–0.20 were left in the model to control for potential confounding.

### Interviews With the Four Surgeons

After surgical training, each surgeon will complete a simple questionnaire. The questionnaire includes the following 7 questions: (1) time for confidence building; (2) conditions for confidence building; (3) the most worried events during the operation; (4) the most worried events during the follow-up; (5) what is the most frustrating thing in the treatment process; (6) list an event that makes you grow fastest; (7) list one of the most critical technologies. Then, we will summarize their questionnaire results.

## Results

### General Results

In 2015–2021, 400 patients were included in the study; all were operated upon by the four surgeons mentioned above. The average patient age was 59.38 ± 21.15 months; 242 were male, 158 were female; and fractures included type II (45.5%) and III (54.5%). The average operation time was 34.00 ± 13.80 min, the average wire placement success rate was 57.75 ± 20.33%, and the anatomical reduction rate was 77%. Of all children, 9.8% required conversion to open reduction and 7.5% required intraoperative superior surgeon guidance. The fractures of all children eventually healed, while 8.8% required more than 2 months to heal. There were no post-operative neurovascular-related complications, while the infection rate was 3.5%. Two infected patients were eventually treated with surgical debridement and healed thereafter. Internal fixation loosening occurred in 12.5% of cases; no other complications occurred. One month after Kirschner wire removal, 81% of the children showed good recovery of elbow function (Table 1).

**Table 1:** Medical and admission characteristics of 400 eligible patient of supracondylar fractures before surgery and subsequent complications.

**General results**		
Age (month)	59.38 ± 21.15	
Sex	Male: 242	39.5%
	Female: 158	60.5%
Side	Left: 230	42.5%
	Right: 170	57.5%
Type	Type II: 182	45.5%
	Type III: 218	54.5%
Accumulation of surgical experience	Before 65: 263	65.8%
	After 65: 137	34.2%
Operation time (min)	34.00 ± 13.80	
Wire placement success rate (%)	57.74 ± 20.33	
Anatomical reduction	Yes: 308	77.0%
	No: 92	23.0%
Conversion to open reduction	Yes: 39	90.3%
	No: 361	9.8%
Intraoperative superior surgeon guidance	Yes: 30	7.5%
	No: 370	92.5%
Infection	Yes: 14	3.5%
	No: 386	96.5%
Internal fixation loosening	Yes: 50	12.5%
	No: 350	87.5%
Delayed union	Yes: 35	8.8%
	No: 365	91.3%
Degree of recovery	Good: 324	81.0%
	Not good: 76	19.0%

### Comparison of Surgeons Background and Operation Experience

The learning experience and internship experience of the four surgeons are shown in Table 2. The average age of the four doctors participating in the training is 27.5 years old, and they are all male. Tow of them have a master's degree and the others have doctor's degrees.

**Table 2:** Surgeons background.

	**Surgeon 1**	**Surgeon 2**	**Surgeon 3**	**Surgeon 4**
Age (y)	28	28	27	27
Sex	Male	Male	Male	Male
Education	Master of Medicine	Master of Medicine	Doctor of Medicine	Doctor of Medicine
Internship experience	General hospital	Children's general hospital	General hospital	Children's general hospital

The four surgeons successfully completed their first 100 supracondylar fractures of humerus during training. Patient information for each surgeon is shown in Table 3. The patients treated by the four surgeons had no significant difference in age (*P* = 0.907), gender (*P* = 0.871), Wire placement success rate (*P* = 0.154), side (*P* = 0.795), Gartland classification (*P* = 0.612), conversion to open reduction (*P* = 0.803), intraoperative guidance (*P* = 0.387), infection (*P* = 0.687), internal fixation loosening (*P* = 0.290)and delayed union (*P* = 0.66). There were differences in operation time and anatomical reduction among the four doctors.

**Table 3:** Patient characteristics for each surgeon.

	**Surgeon 1**	**Surgeon 2**	**Surgeon 3**	**Surgeon 4**	***P-*value**
**Sex (** ***n*** **)**				
Male	62	57	62	61	0.871
Female	38	43	38	39	
Age	60.2 ± 26.93	59.4 ± 24.32	60.44 ± 20.41	57.72 ± 30.4	0.907
**Side (** ***n*** **)**					
Left	57	54	61	58	0.795
Right	43	46	39	42	
**Type (** ***n*** **)**					
Type II	49	46	40	47	0.612
Type III	51	54	60	53	
Operation time (min)	36.54 ± 14.01	37.48 ± 12.84	29.32 ± 13.75	32.66 ± 13.21	<0.01
Wire placement success rate (%)	56.92 ± 20.72	56.58 ± 18.64	55.9 ± 22.27	61.57 ± 19.3	0.183
Anatomical reduction (*n*)	72	77	71	88	0.021
Conversion to open reduction (*n*)	9	10	12	8	0.803
Intraoperative superior surgeon guidance (*n*)	10	7	9	4	0.387
Infection (*n*)	5	4	2	3	0.687
Internal fixation loosening (*n*)	8	13	17	12	0.29
Delayed union (*n*)	7	10	11	7	0.66

### Regression Splines of Relationship of Operation Time and Wire Placement Success Rate

**Figure 1:**
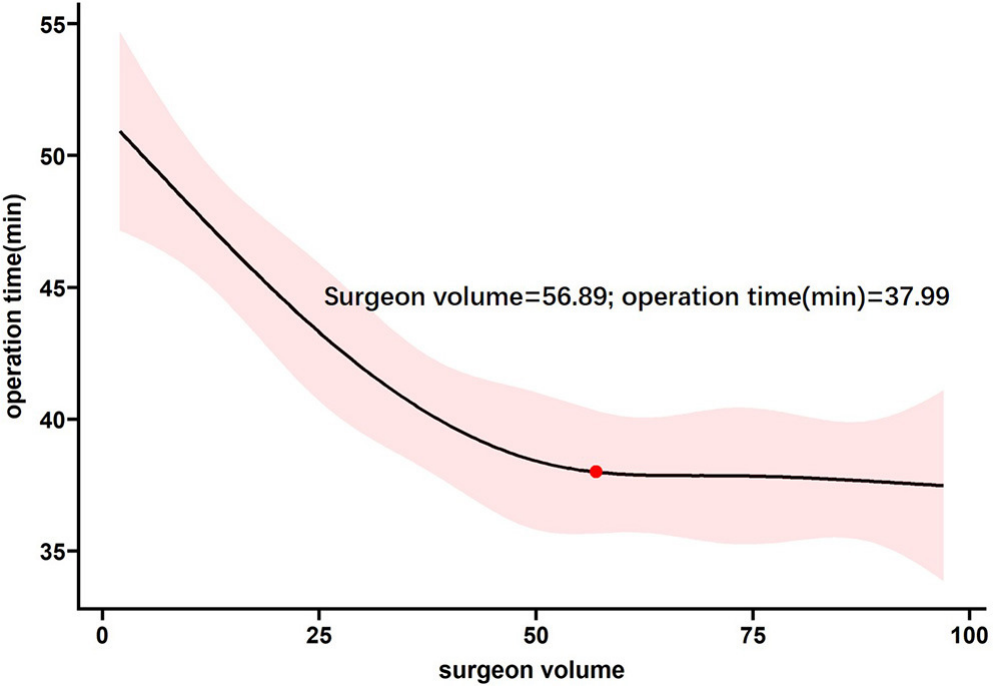
Probability of operation time for supracondylar fractures according to surgeon volume.

**Figure 2:**
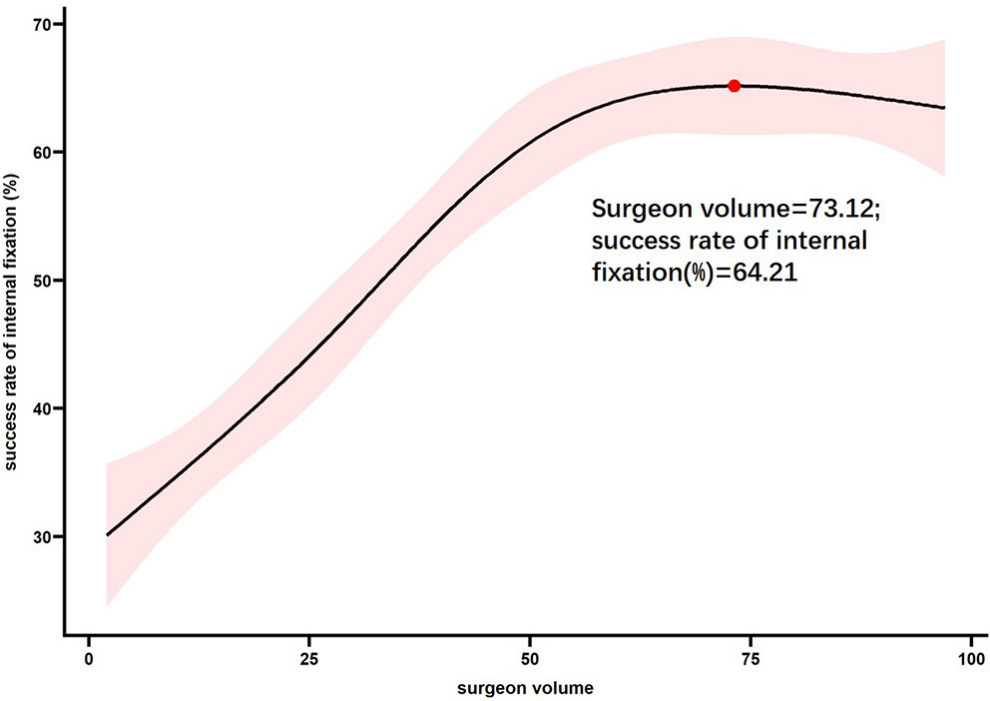
Probability of success rate of internal fixation according to surgeon volume.

The restricted cubic splines for surgical experience to time and rate had similar inflection points at ~65 procedures, after which point, with the continued accumulation of surgical experience, the trend of increasing operation time and decreasing success rate flattened (Figures 1, 2).

We dichotomized the surgical experience according to a volume of 65 procedures (≤65 or >65). Before and after the accumulation of surgical experience, the two groups of patients were compared in terms of age (58.19 ± 25.18 vs. 61.66 ± 30.54 months), sex (male, 154 vs. 88; female, 109 vs. 49), and classification (type II, 139 vs. 79; type III, 124 vs. 58). In terms of post-operative evaluation, the operation time, anatomical reduction (186 vs. 122, *P* < 0.001), conversion to incision (33 vs. 6, *P* = 0.008), and superior physician guidance (28 vs. 2, *P* < 0.001) were significantly different. Regarding post-operative complications, there was no significant difference in infection (12 vs. 2, *P* = 0.153) or delayed union (28 vs. 7, *P* = 0.065). There were significant differences in internal fixation loosening (41 vs. 9, *P* = 0.01) and poor post-operative functional recovery (67 vs. 9, *P* < 0.001) (Table 4).

**Table 4:** Comparison of characteristics of patient, before, and after accumulation of surgical experience.

**Results**	**Before 65**	**After 65**	***P-*value**
**Sex (** ***n*** **)**			
Male	154	88	*P* = 0.238
Female	109	49	
Age	58.19 ± 25.18	61.66 ± 30.54	*P* = 0.225
**Side (** ***n*** **)**			
Left	153	77	*P* = 0.749
Right	110	60	
**Type (** ***n*** **)**			
Type II	124	58	*P* = 0.398
Type III	139	79	
Operation time (min)	35.39 ± 15.05	31.32 ± 10.59	*P* = 0.005
Wire placement success rate (%)	52.74 ± 19.76	67.34 ± 17.87	*P* < 0.001
**Anatomical reduction**			
Yes	186	122	*P* < 0.001
No	77	15	
Conversion to open reduction (*n*)			*P* = 0.008
Yes	33	6	
No	230	131	
**Intraoperative superior surgeon guidance (** ***n*** **)**			
Yes	28	2	*P* < 0.001
No	235	135	
**Infection (** ***n*** **)**			
Yes	12	2	*P* = 0.153
No	251	135	
**Internal fixation loosening (** ***n*** **)**			
Yes	41	9	*P* = 0.01
No	222	128	
**Delayed union (** ***n*** **)**			
Yes	28	7	*P* = 0.065
No	235	130	
**Good functional recovery (** ***n*** **)**			
Yes	196	128	*P* = 0.001
No	67	9	

### Multivariate Logistic Regression Analysis of Post-operative Functional Recovery

Subsequently, we conducted analysis of variance with the accumulation of surgical experience as an independent risk factor affecting post-operative functional recovery and found that the effect of surgical experience was significant. Similarly, an analysis of variance was performed for other continuous variables (age, duration of surgery, and wire placement success rate). The chi-square test was performed of the risk factors of the dichotomous variables, and the results showed no difference between the sexes, affected side, or type. However, significant differences were observed in conversion to open reduction, infection, and delayed healing (Table 5).

**Table 5:** Univariate analysis of functional recovery in eligible patient.

	**Recovery**		**OR**	***P-*value**
	**Good**	**Not good**		
Operation time (min)	32.86 ± 12.61	38.85 ± 17.28		<0.001
Wire placement success rate (%)	59.04 ± 20.51	52.20 ± 18.67		0.005
Age (month)	71.84 ± 28.91	60.03 ± 23.19		0.001
**Sex**				
Male	193	49	Female/male = 0.812	0.515
Female	131	27	CI: 0.483–1.365	
**Side**				
Right	134	36	Left/right = 1.276	0.368
Left	190	40	CI: 0.773–2.107	
**Type**				
Type II	151	31	Type II/Type III = 0.789	0.373
Type III	173	45	CI: 0.475–1.310	
**Surgical experience**				
<65	197	66	Before 65/after 65 = 4.255	<0.001
>65	127	10	CI: 2.110–8.581	
**Anatomical reduction**				
Yes	259	49	0/1 = 2.196	0.006
No	65	27	CI: 1.276–3.778	
**Conversion to open reduction (** ***n*** **)**				
Yes	22	17	0/1 = 0.253	<0.001
No	302	59	CI: 0.127–0.505	
**Intraoperative superior surgeon guidance (** ***n*** **)**				
Yes	22	8	0/1 = 0.619	0.330
No	302	68	CI: 0.261–1.450	
**Infection (** ***n*** **)**				
Yes	8	6	0/1 = 0.295	0.032
no	316	70	CI: 0.099–0.878	
**Internal fixation loosening (** ***n*** **)**				
Yes	38	12	0/1 = 0.709	0.338
No	286	64	CI: 0.351–1.432	
**Delayed union (** ***n*** **)**				
Yes	23	12	0/1 = 0.408	0.023
No	301	64	CI: 0.193–0.861	

After excluding risk factors with values of *P* > 0.2 (type, side, intraoperative guidance, and implant loosening), we performed multivariate logistic regression of post-operative functional recovery. Surgical experience, infection, and conversion to incision significantly affected the prognosis of closed reduction and internal fixation of supracondylar fractures of the humerus (Table 6).

**Table 6:** Multivariate logistic regression analysis of post-operative functional recovery in eligible patient.

	**Good**	**Not good**	***P-*value**
Age (month)	71.84 ± 28.91	60.03 ± 23.19	0.448
Operation time (min)	32.86 ± 12.61	38.85 ± 17.28	0.344
Wire placement success rate (%)	59.04 ± 20.51	52.20 ± 18.67	0.831
**Surgical experience**			
<65	197	66	0.001
>65	127	10	
**Anatomical reduction**			
Yes	259	49	0.456
No	65	27	
**Conversion to open reduction (** ***n*** **)**			
Yes	22	17	0.025
No	302	59	
**Infection (** ***n*** **)**			
Yes	8	6	0.042
No	316	70	
**Delayed union (** ***n*** **)**			
Yes	23	12	0.343
No	301	64	

### Interviews With the Four Surgeons and One Supervisor

We were fortunate to receive the response from four surgeons and one of the oldest supervisors. As shown in Table 7, the interviewees' answers to most questions have something in common: (1) it usually takes about 3 months to establish surgical self-confidence (about 30 supracondylar fractures); (2) the opportunity to establish self-confidence is usually to successfully complete difficult surgery independently; (3) the surgeons are most worried about the difficulty of intraoperative reduction and post-operative complications; (4) the most frustrating thing is the poor functional recovery of patients after operation; (5) manual traction and closed reduction is considered to be the most critical surgical technique.

**Table 7:** Interview contents after the training.

**Supervisor**	**Surgeon 4**	**Surgeon 3**	**Surgeon 2**	**Surgeon 1**	
3 months 30 operations	15–20 operations	3 months 34 operations	2–3 months 21–30 operations	About 2 months 22 operations	Time for confidence building
Operation completed within 30 min	Continuous successful operation	Successful reduction of irreducible supracondylar fracture	After completing some difficult operations independently	Presence of Supervisor	Conditions for confidence building
The patient was over 10 years old with obvious swelling	Fracture reduction failed without supervisor's help	Open reduction failure •Need intraoperative guidance	• Repeated fracture reduction failure • Iatrogenic neurovascular injury	Iatrogenic neurovascular injury	The most worried events during the operation
Neurologic complications	Various post-operative complications	Infection	• Fracture displacement • Infection	Infection	The most worried events during the follow-up
Transfer to open reduction	Poor functional recovery in case of good reduction, fixation and healing	Infection	Poor functional recovery due to anatomical reduction failure	Poor recovery of elbow function	What is the most frustrating thing in the treatment process
Solo the operations without any assistant	Successful operation of first flexion supracondylar fracture	Hands on guidance of supervisors	Successful operation of first flexion supracondylar fracture	Success of the first open reduction and internal fixation	List an event that makes you grow fastest
Close reduction	Close reduction	Theoretical basis of supracondylar fracture	Percutaneous K-wire fixation	Close reduction	List one of the most critical technologies

## Discussion

RCS is a commonly used method to explain the non-linear relationship between variables and outcomes, whether it is the research on disease mechanism (10, 11), treatment methods (12), health management (13) or hospital management (14). To our knowledge, this study is the first application of RCS in surgical learning curve.

Supracondylar fractures of the humerus are suitable candidates for fresh pediatric orthopedists because the treatment of this disease is very mature and it involves a standardized diagnosis and treatment process (6, 15–17). Just as a pilot learns to fly a plane, he must start with the most productive, most commonly used, and safest aircraft. The treatment options for supracondylar fractures of the humerus were described in detail in a 1997 review by Otsuka and Kasser (7). Closed reduction and internal fixation of fractures are suitable for most patients with type II and III fractures, and it is already a very mature treatment plan. For the four surgeons in this study, there were no differences in diagnosis or treatment plan (all patients were treated after discussions with all doctors in the department). The center did not allow these four surgeons to perform operations that required neurovascular exploration, which would involve more severe fractures, the intraoperative operation review would be more complex, and the learning curve would be steeper, an unsuitable course for beginners.

Following the outcomes of the cases in the study, we believe that supracondylar fracture of the humerus was an appropriate disease for studying the learning curve, although for these 400 children, our department always provided them with senior orthopedic physicians on duty in case rescue became necessary. However, the research process was safe and no accidents occurred. The 400 patients included in this study had no neurovascular complications or requirement for revision after delayed union. There were patients with loose fixation, but none required re-fixation. There was one case of infection, but it did not require ongoing treatment. Thus, closed reduction and internal fixation with a Kirschner wire for supracondylar fractures of the humerus is a safe, effective, and suitable surgical technique for beginners.

The children in this study were equally distributed to each suregon in age, sex and classification. This suggests that our study population did not suffer from selection bias. Through the RCS fitting curve of the number of surgical cases, operation time, and success rate of wire placement, the operation time and wire placement success rate were moderated in the plateau period after the accumulation of experience with 60–70 procedures. Therefore, we considered the experience accumulation of 65 operations as the inflection point for supracondylar fracture surgeries. We also compared data on surgical technique and post-operative prognosis before and after the 65 operations and found statistically significant differences in operation time, wire placement success rate, and converted to open reduction, indicating that the accumulation of experience in 65 operations was reasonable.

Multiple logistic regression analysis revealed that, although the operation time and wire placement success rate included in the RCS mapping could reflect accumulation of surgeon experience, they did not affect post-operative functional recovery. Similarly, our study found that the guidance of supervising surgeons did not affect post-operative recovery. Before obtaining the help of supervising surgeons, the intraoperative operation of fresh resident surgeons may include multiple steps such as disinfection, traction reduction, and internal fixation with a Kirschner wire. However, the inability to complete the reduction or experience of several failed attempts at implant placement did not affect patient prognosis. This conclusion can also increase fresh surgeon confidence.

Loosening of the internal fixation, a common complication of percutaneous Kirschner wire fixation, had no effect on post-operative functional recovery. The usual incidence was about 5–8%, and our incidence was 12%, which was slightly higher than that reported by other centers (18, 19). This may be related to the novice's proficiency at mastering internal fixation. In addition, we also found that loosening of internal fixation may be more common in fractures without anatomical reduction. Because the position of K-wire shown on the anteroposterior and lateral radiographs of the fracture that did not achieve anatomical reduction may be false, misleading the surgeons to confirm the position, resulting in the loosening. The author suggests that when anatomical reduction cannot be achieved, taking multi angle radiography can help the surgeons better determine the position of K-wire.

The accumulation of surgical experience, intraoperative conversion to open reduction, and post-operative infection were risk factors affecting functional recovery after surgery. Intraoperative conversion to open reduction often indicates that the fracture is difficult to reduce and unstable and that the periosteum is severely torn. Moreover, the broken ends of fractures are usually embedded in soft tissues such as muscles, blood vessels, and nerves. Moreover, in the process of open reduction, the soft tissue near the fracture will be destroyed iatrogenically, further causing post-operative recovery difficulties. Therefore, the use of open reduction should be reduced as much as possible. At present, supracondylar fractures of the humerus increasingly require open reduction, and many reports have reported reduction methods for refractory supracondylar fractures (20, 21). However, these are advanced techniques for fracture reduction that are difficult for beginner to master. Combined with this study's findings, we found that anatomical reduction was not a risk factor for poor prognosis. Therefore, blindly pursuing anatomical reduction may not be necessary for fresh surgeons.

Infection, a common complication that affects the prognosis of all fractures, is divided into intraoperative and post-operative. Intraoperative infection requires attention to intraoperative aseptic technique. It has been suggested that surgical procedures can be performed after local disinfection without increased infection rates, which may be possible for experienced physicians. However, for new surgeons, we still recommend that they strictly grasp the concept of aseptic technique to avoid intraoperative infection. In this study, the infection eventually progressed to osteomyelitis in two patients who underwent debridement. During the debridement operation, we found that the source of infection was the part where the tail of the wire contacted the skin. This reminds us that guidance is also required for handling K-wire tails. In future teaching, we must remember to emphasize the importance of infection control.

**Figure 3:**
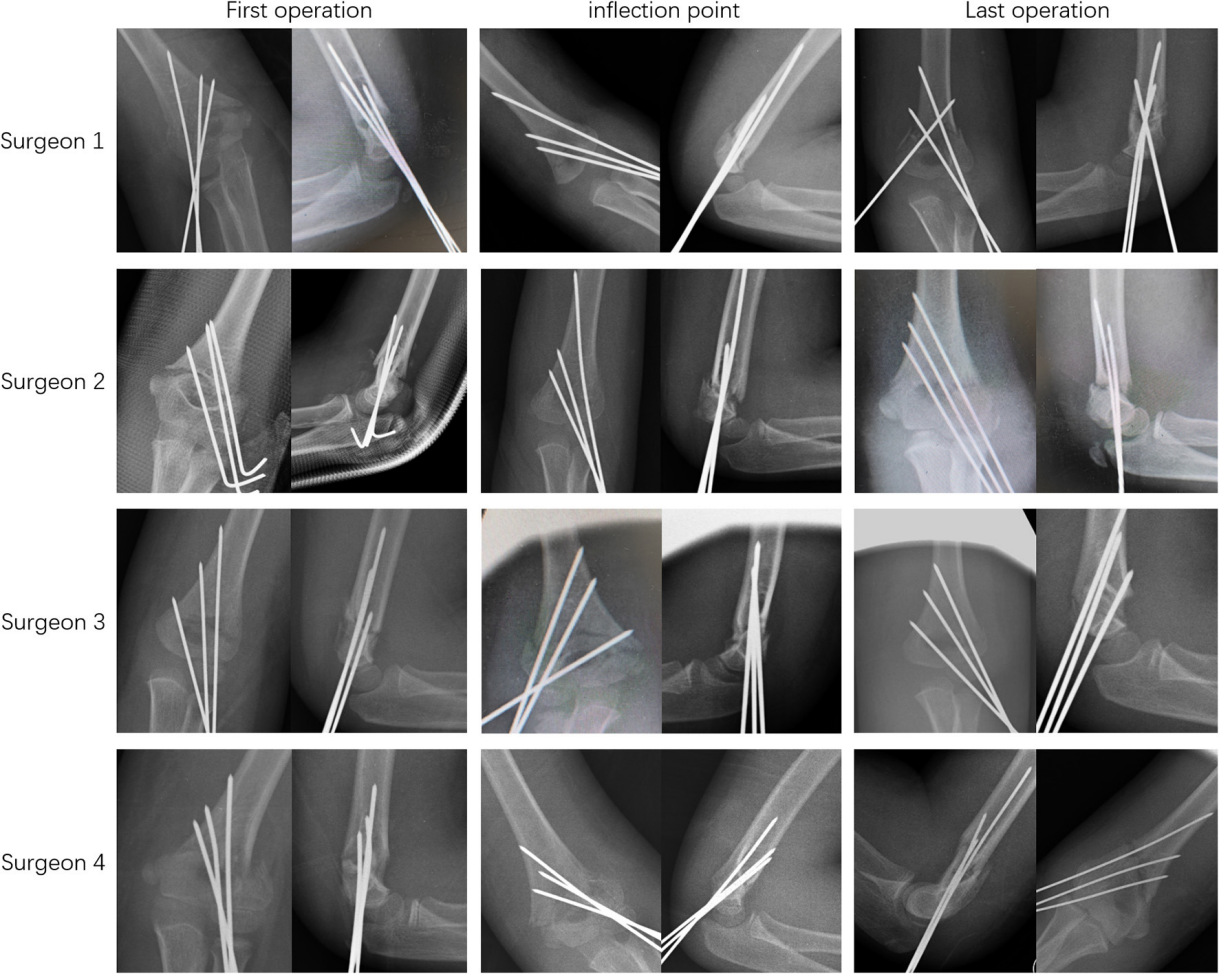
Progress in surgical skills of the four surgeons: Both the fracture reduction and K-wire arrangement of the four surgeons tend to be perfect with the accumulation of surgical experience.

As shown in Figure 3, we may clearly see the progress of the four surgeons. The experience accumulation of 65 operations was beyond our expectations. From our data, we can find that earlier than 65 operations, our four fresh surgeons have rarely needed supervisors to guide the operation. Therefore, we believe that the experience accumulation of 65 operations is not the time required to learn one operation, but the time required to achieve the stability of personal surgical techniques. Just like the arthroscopic learning curve in the literature you provided, the experience accumulation of 170 surgeries can reach the level of consultant (5). Our experience in 65 operations may not be learning a technique, but mastering a technique.

In our interview, we found that the accumulation of experience required by fresh surgeons to build their confidence is generally less than the inflection point obtained in this study. But this does not mean that the calculation of our inflection point is wrong. Every operation has key techniques that can be perceived by the trainer and those that cannot be perceived. This key technology that cannot be perceived can be called “hidden points”. Fracture reduction, for example, is a key perceptible technique. This has attracted the attention of our center. Teachers will focus on the guidance of key technologies mentioned in the questionnaire.

However, the imperceptible key technology may exist in theory, skills, psychology and so on. So we invited a psychologist to describe the characters of the five respondents only based on their questionnaires, and provided them psychological guidance. We believe that if psychological intervention is regularly added in the process of surgical training, it will be more conducive to the growth of young doctors (Supplementary Material).

In the subsequent resident training, we will pay more attention to the number and time of the supervising doctor's guidance when the resident is an assistant. We have added an additional “preparatory period” of 6 months for fresh surgeons. During the preparatory period, our fresh surgeons will follow supervising doctors on duty in the entire ward the next day to complete emergency operations. Intraoperatively, the fresh one learns to operate in the position of chief surgeon, but the supervising doctor must function as an assistant. According to the total number of operations in our center, two fresh surgeons can each participate in treating ~60 humerus supracondylar fractures in 4 months of training, and the amount of training in the preparatory period has reached the learning curve required for this study. In a follow-up study, we will compare the performance of new orthopedic surgeons after the adjustment for the training method.

However, there was still a lack of attention to patients in this study. We found that operations with an operation time >90 min occurred before the accumulation of experience with 65 units. Therefore, we added the indicators of active intervention by the superior surgeons in the following medical arrangements: (1) existing combined neurovascular injury and suspected compartment syndrome; (2) irreducible supracondylar fracture of the humerus; and (3) surgery time >60 min. This can avoid excessive damage to the patient without putting pressure on new surgeons, although the time of the operation does not affect the surgical prognosis. In this way, we can shorten the time limit for higher-level surgeons to prepare shifts from 1 year to half a year, which greatly saves medical resources and affords patients better treatment.

## Conclusion

Although fresh surgeons can establish self-confidence in surgery through 30 operations, we still insist on accumulating experience in 65 operations to master closed reduction and internal fixation of supracondylar fractures. The accumulation of surgical experience, infection and conversion to open reduction are the risk factors affecting the recovery of elbow function. Strengthening the supervisor's guidance on the key techniques and regular psychological guidance to find hidden point may help fresh surgeons master the surgical techniques as soon as possible.

## Data Availability Statement

The original contributions presented in the study are included in the article/Supplementary Material, further inquiries can be directed to the corresponding authors.

## Author Contributions

CQ and YZ: operations, data collection, performed measurements, and manuscript preparation. JM and YM: operations, data collection, and performed measurements. JS: psychological evaluation. HL: study design, statistical analysis, and manuscript preparation. DW: study design and manuscript preparation.

## Conflict of Interest

The authors declare that the research was conducted in the absence of any commercial or financial relationships that could be construed as a potential conflict of interest.

## Publisher's Note

All claims expressed in this article are solely those of the authors and do not necessarily represent those of their affiliated organizations, or those of the publisher, the editors and the reviewers. Any product that may be evaluated in this article, or claim that may be made by its manufacturer, is not guaranteed or endorsed by the publisher.
