# Supplementary material for: Learning Curve of Closed Reduction and Internal Fixation for Supracondylar Fractures of the Humerus in Children
Source: Front Pediatr. 2022 Jul 7;10:945616. doi: 10.3389/fped.2022.945616 (PMC9301003; doi:10.3389/fped.2022.945616)
Supplement: Supplementary file 1 [file Data_Sheet_1.DOCX]

supplement 1. Character description and psychological guidance

**Surgeon 1**

**Character description**

With the support of supervisors, he dares to explore and try after early training. When he completed an operation independently for the first time, he would be anxious and nervous, but he finished the operation without danger. This will promote his sense of achievement and confidence in similar operations in the future.

**Psychological guidance**

We need to pay attention to how to adjust his surgical preparation and self pressure in the future without a supervisor, so as to meet new challenges.

**Surgeon 2**

**Character description**

Although he has many successful experience in completing the operation, he is still under pressure to deal with the key techniques in the new operation. He showed a good sense of professional responsibility and professionalism in the training process.

**Psychological guidance**

He needs to constantly have new career growth happiness points and continue to improve his sense of self-efficacy. This can help him have a more mature psychology when dealing with complex surgery in the future.

**Surgeon 3**

**Character description**

In the training process, he has his own anxiety and the pressure brought by the operation itself. Fortunately, he can always get the guidance of his superior doctor and relieve the pressure. At this time, he can well feel the power of the group. He will take the initiative to find his own resources and have a strong sense of collaboration.

**Psychological guidance**

In the future, he needs to learn how to face the pressure independently. How to prepare and deal with emotional tension when he needs to operate independently. It will be very helpful for him to ask the superior doctors to share their successful experience in the process of growth.

**Surgeon 4**

**Character description**

As an surgeon who has successfully completed many operations in succession, his experience has been internalized into a sense of professional honor, self-confidence and good self-efficacy. However, his sense of professional responsibility still plays a role at this time. He can pay attention to the risks of surgery, prevent and avoid worrying events, and successfully deal with various challenges.

**Psychological guidance**

In the future, he can pay more attention to the cultivation of professional responsibility to become an excellent surgeon with high comprehensive quality.

**Supervisor**

**Character description**

As an surgeon who can skillfully complete the operation, as well as a teacher, he is able to cope with the psychological pressure of the operation itself, and can also set a good example for novices. It relies on his experience, skills and confidence in solo operations.

**Psychological guidance**

If he can continuously improve his sense of professional responsibility and good psychological quality, he will become an excellent surgeon and an excellent teacher. However, because he enjoys the feeling of completing solo operations, he needs to learn psychological adjustment and strengthen relaxation training.
